# Transfemoral transcatheter aortic valve-in-valve implantation using endoconduit in a patient with right-sided aortic arch and complex vascular anatomy: a case report

**DOI:** 10.1093/ehjcr/ytaf315

**Published:** 2025-06-30

**Authors:** Ryo Otake, Kazuki Mizutani, Ryo Horita, Umihiko Kaneko, Daisuke Hachinohe

**Affiliations:** Department of Cardiology, Asia Medical Group, Sapporo Heart Center, Sapporo Cardio Vascular Clinic, North 49, East 16, 8-1, Higashi Ward, Sapporo, Hokkaido 007-0849, Japan; Department of Cardiology, Asia Medical Group, Sapporo Heart Center, Sapporo Cardio Vascular Clinic, North 49, East 16, 8-1, Higashi Ward, Sapporo, Hokkaido 007-0849, Japan; Department of Cardiology, Asia Medical Group, Sapporo Heart Center, Sapporo Cardio Vascular Clinic, North 49, East 16, 8-1, Higashi Ward, Sapporo, Hokkaido 007-0849, Japan; Department of Cardiology, Asia Medical Group, Sapporo Heart Center, Sapporo Cardio Vascular Clinic, North 49, East 16, 8-1, Higashi Ward, Sapporo, Hokkaido 007-0849, Japan; Department of Cardiology, Asia Medical Group, Sapporo Heart Center, Sapporo Cardio Vascular Clinic, North 49, East 16, 8-1, Higashi Ward, Sapporo, Hokkaido 007-0849, Japan

**Keywords:** Aortic stenosis, Aortic regurgitation, Right-sided aortic arch, Structural valve deterioration, Transcatheter aortic valve implantation, Endoconduit, Case report

## Abstract

**Background:**

The number of transcatheter aortic valve implantation (TAVI) procedures has increased significantly and now includes younger and higher-risk surgical patients. As early-generation transcatheter heart valves (THVs) continue to degrade over time, the incidence of structural valve deterioration (SVD) is increasing, requiring more complex valve-in-valve (TAV-in-TAV) procedures.

**Case summary:**

We present a case of SVD of a THV, resulting in severe aortic regurgitation in an 88-year-old female with decompensated heart failure. Eight years prior, the patient had undergone TAVI with SAPIEN XT 23 mm via the transapical approach because of severe calcified stenosis of the lower extremity arteries and extreme tortuosity from the arch to the descending aorta and abdominal aortic aneurysm, right-sided aortic arch. Considering the patient’s condition and access route, we performed emergency transfemoral TAV-in-TAV using a 23 mm SAPIEN3 Ultra RESILIA. The endoconduit technique successfully facilitated sheath passage. The valve was implanted without complications. The procedure was successful, resulting in only trace paravalvular regurgitation. The post-operative effective orifice area was 1.80 cm² (indexed EOA: 1.32 cm²/m²). The Doppler Velocity Index was 0.59, suggesting no significant patient–prosthesis mismatch.

**Discussion:**

Vascular anomalies often restrict the choice of a safe access route in TAVI procedures. The endoconduit technique provides a minimally invasive solution for accessing complex iliac arteries. This case illustrates the importance of selecting the appropriate sheath, utilising endoconduit, and employing various procedural techniques to manage complex anatomies and ensure successful outcomes in TAV-in-TAV procedures.

Learning points

- As structural valve deterioration of early-generation THV increases, the need for TAV-in-TAV procedures is rising. Patients who underwent the initial TAVI via an alternative approach often have challenging anatomy, requiring more complex techniques.
- In TAV-in-TAV procedures, maintaining an effective orifice area >1.5 cm² is important to reduce the risk of patient-prosthesis mismatch and ensure favourable haemodynamic outcomes.

## Introduction

Transcatheter aortic valve-in-valve (TAV-in-TAV) implantation is effective for patients with structural valve deterioration (SVD), achieving a lower 30-day mortality rate than redo surgical aortic valve replacement.^1^ Additionally, Transcatheter Aortic Valve Implantation (TAVI) has demonstrated favourable outcomes compared with surgical procedures.^2^ Consequently, the number of TAVI procedures has increased,^3^ presenting new challenges as the procedure expands to younger patients with higher surgical risks. Conversely, most patients with SVD are treated with early-generation transcatheter heart valves (THV), at high surgical risk owing to initial TAVI. These patients often present with more complex clinical features and poorer vascular access than at initial treatment, making it necessary to devise safe methods for TAV-in-TAV.

## Summary figure

**Table:** 

Time	Event
8 years before admission	Transapical TAVI with a 23-mm SAPIEN XT (Edwards Lifesciences, Irvine, CA, USA).
Time of admission	Admitted with decompensated heart failure (New York Heart Association Functional Classification III) and severe transvalvular regurgitation.
Day 1 to Day 5 after admission	Despite heart failure management with furosemide, low dose dobutamine, and dialysis, the patient remained in shock. Continuous hemodiafiltration was initiated, but her condition stayed critical.
Day 5 after admission	Heart team decision to perform urgent TAV-in-TAV based on clinical frailty score of 5, Society of Thoracic Surgeons score of 31.2%.
Day 6 after admission	Transfemoral TAV-in-TAV procedure performed with a 20-mm SAPIEN3 Ultra RESILIA (Edwards Lifesciences, Irvine, CA, USA).
Day 6 after procedure	Move to the general ward.
Day 53 After Procedure	Changed To A Rehabilitation Hospital. An Mavpg Of 12 Mmhg And An EOA Of 1.80 Cm^2^ At Discharge.

## Case presentation

An 88-year-old woman with chronic atrial fibrillation and stage 3b chronic kidney disease accompanied by decompensated heart failure (New York Heart Association Functional Classification III) was transferred to our hospital. Eight years prior, she underwent TAVI with a 23 mm SAPIEN XT (Edwards Lifesciences) via the transapical (TA) approach due to a right-sided aortic arch (RSAA) and severe calcified stenosis of the bilateral iliac arteries.

Echocardiography revealed preserved left ventricular ejection fraction and severe transvalvular regurgitation (jet deviated from the non-coronary cusp side; vena contracta 6.2 mm; pressure half-time 204 ms), along with elevated estimated right ventricular pressure and severe tricuspid regurgitation. Her blood pressure was 108/52 mmHg, pulse rate 96 bpm, respiratory rate 25 breaths per minute, and oxygen saturation 92% on room air. Physical examination revealed a Levine grade 4/6 to-and-fro murmur, best heard at the left third intercostal space along the left sternal border. In addition, jugular venous distension was noted, along with bilateral lower extremity oedema and fine crackles in both lung fields, suggesting volume overload. Endocarditis was excluded by negative blood cultures, clinical assessment, and echocardiographic findings. Despite intravenous furosemide and low-dose dobutamine (2 μg/kg/min), heart failure did not improve. Continuous hemodiafiltration was initiated; however, her blood pressure decreased, leading to shock. Urgent TAV-in-TAV was performed, given her clinical frailty score of 5 and Society of Thoracic Surgeons score of 31.2%. Contrast-enhanced computed tomography showed mild leaflet thickening, an annulus area of 338.0 mm^2^, a perimeter of 76.6 mm, and an inner diameter of 19.7 × 21.9 mm. The sinus of Valsalva and sinotubular junction were sufficiently large, and the risk plane was located below each coronary artery. The distance from the transcatheter valve to each coronary artery was >4 mm, indicating a low risk of coronary occlusion.

**Figure 1:**
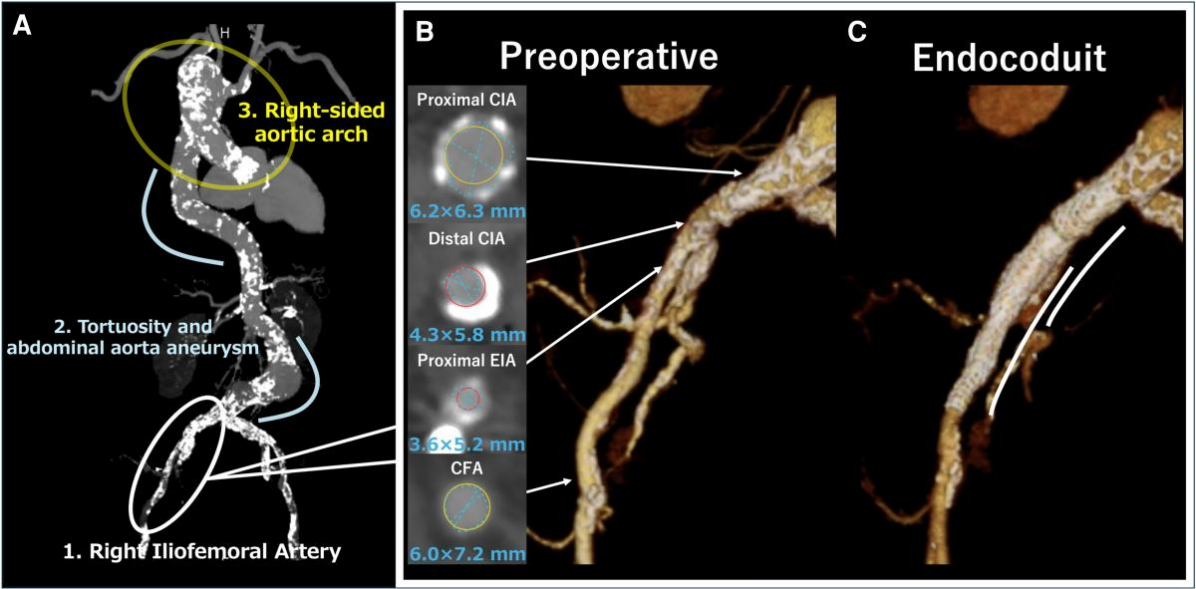
(*A*) Computed tomography angiography showing three major anatomical considerations for TAV-in-TAV. (*B*) Preoperative image of the iliofemoral arterial anatomy. Cross-sectional images showing measurements at various key points. (*C*) Post-operative image after the endoconduit procedure.

Three concerns were noted when selecting TF (*Figure 1A*).

1. The severely calcified common iliac artery (CIA) measured, external iliac artery (EIA) measured, and common femoral artery (CFA) measured 6.2 × 6.3, 3.6 × 5.2, and 6.0 × 7.2 mm, respectively. Severe calcification was noted from the EIA to CIA (*Figure 1B*).
2. Extreme tortuosity from the arch to the descending aorta and abdominal aortic aneurysm was observed.
3. The patient had an RSAA with an aberrant left subclavian artery.

Alternative TAVI access routes, including TA, transaortic (TAo), transsubclavian, transcarotid, transcaval, and suprasternal approaches, were evaluated, each with unique advantages and limitations dictated by patient anatomy and clinical context.^4^ The transcaval approach was excluded due to severe aortic tortuosity and abdominal aortic aneurysm. Subclavian, carotid, and suprasternal approaches were unsuitable due to vascular calcifications and anatomical challenges from the RSAA. Due to the patient’s frailty, TA and TAo were excluded due to the higher perioperative risks and required general anaesthesia. Finally, TF was selected as the most suitable and least invasive option.

**Figure 2:**
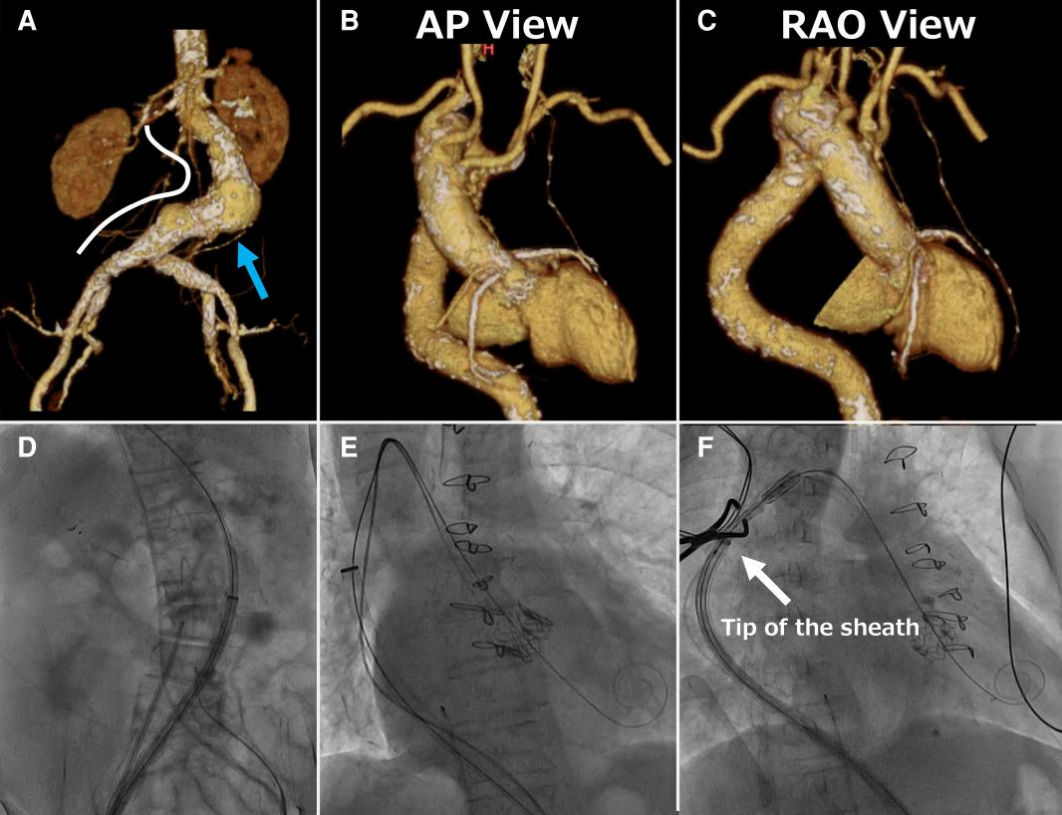
A and D: (*A*) CT volume rendering showing an abdominal aortic aneurysm (arrow) with severe tortuosity and (*D*) the corresponding fluoroscopic view. B and E: (*B*) Anteroposterior view of CT volume rendering and (*E*) the corresponding fluoroscopic view. C and F: (*D*) Right anterior oblique view of the CT volume rendering and (*F*) corresponding fluoroscopic view. CT, Computed tomography.

**Figure 3:**
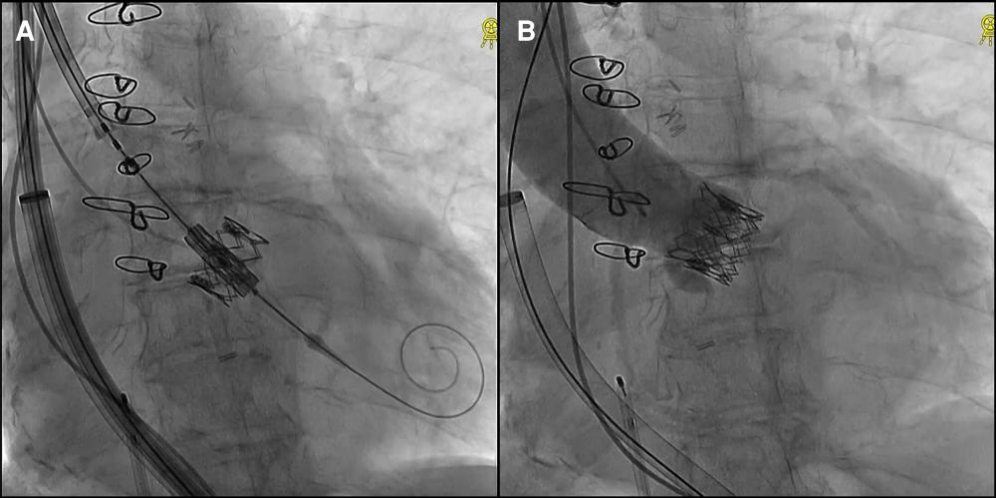
TAV-in-TAV with 23 mm SAPIEN3 ultra RESILIA. (*A*) Fluoroscopic view before TAV-in-TAV. (*B*) Aortogram of the TAV-in-TAV. TAV-in-TAV, Transcatheter aortic valve-in-valve.

We selected the SAPIEN 3 Ultra RESILIA 23 mm (Edwards Lifesciences) due to its superior stability and precise control during deployment, which was critical in this complex anatomy with severe tortuosity and an RSAA. In addition, we used the Commander delivery system with flex capability to navigate the tortuous aorta, advancing the valve through a 20 Fr DrySeal sheath (W.L. Gore & Associates) with an effective length of 64 cm. Despite attempts to use a stiffer guidewire, the sheath could not pass through the distal EIA. Serial balloon pre-dilatation with 8.0 × 40 mm and 10.0 × 20 mm non-compliant balloons (Mustang, Boston Scientific) at 14 atm did not facilitate successful passage. Deployment of 10.0 × 58 mm Lifestream stent grafts (BD, Franklin Lakes) in the distal CIA and EIA was required (*Figure 1C*). Further angiography revealed no bleeding, confirming successful sheath passage (see Supplementary material online, *Video S1*). Despite extreme tortuosity and abdominal aortic aneurysm, the DrySeal sheath successfully passed through without complications (*Figure 2A* and *D*). During valve advancement, the right anterior oblique view enhanced RSAA visualisation. The DrySeal sheath was advanced to the distal arch to enhance backup support and facilitate passage through the RSAA (*Figure 2B, C, E* and *F*, Supplementary material online, *Video S2*). Careful positioning was performed to align the lower ends of each valve to ensure optimal implantation (*Figure 3A*). Post-dilatation was performed to ensure suboptimal expansion of the valve at the same pressure used during valve implantation. Final angiography revealed minimal aortic regurgitation (*Figure 3B*; Supplementary material online, *Video S2*). Post-operative echocardiography confirmed good prosthetic valve function, with a mean aortic valve pressure gradient of 12 mmHg, an effective orifice area (EOA) of 1.80 cm^2^ (index EOA: 1.32 cm^2^/m^2^), and trace paravalvular leakage. Additionally, the Doppler Velocity Index was 0.59, indicating no significant patient-prosthesis mismatch. The patient was successfully weaned off catecholamines and discharged on day 6 without any other major complications.

## Discussion

In TAVI procedures, selecting a safe access route is often challenging due to severe vascular calcification, tortuosity, stenosis, or vascular anomalies. Patients implanted with early-generation THV and subsequent indications for TAV-in-TAV have a high surgical risk and often have poor vascular access. Additionally, the presence of an RSAA (incidence rate of 0.05%) presents unique challenges due to its rarity.^5^

Successful TAVI using a 26 mm Evolut-FX valve with a 14 French-compatible InLine sheath (Medtronic) has been reported in cases with RSAA and significant aortic tortuosity.^6^ Self-expanding valves such as the Evolut-FX are known to provide lower transvalvular pressure gradients, which is beneficial for patients with smaller annuli and higher baseline gradients. However, in challenging cases such as ours, with RSAA and extremely poor vascular conditions, we selected the Sapien 3 Ultra RESILIA with the Commander system for its advantageous flex mechanism. Given the eSheath was too short, we selected a DrySeal sheath with an effective length of 64 cm, which provided adequate backup. This combination of the Commander system and a long sheath is the optimal strategy for such challenging cases. Additionally, we carefully adjusted the rotation of the delivery system under fluoroscopy to avoid contact with the aorta. In cases with RSAA, if the patient's anatomical structure is ‘reversed’, it may be necessary to rotate the delivery system 180°.^7^

In the treatment of severely calcified iliac artery lesions, intravascular lithotripsy (IVL) is considered an effective and minimally invasive option. In similar cases, IVL is generally preferred as the initial strategy due to its simplicity and minimal invasiveness, with the endoconduit technique reserved for cases where sheath advancement remains unsuccessful. However, IVL is not currently approved for use in lower extremity arteries in our region and therefore was not an option in this case. The Lifestream stent graft was selected for the endoconduit technique because its interconnected links and minimal shortening provide excellent longitudinal stability and facilitate vessel straightening, both of which are essential for advancing the sheath through severely calcified arteries. Although alternatives such as the VIABAHN VBX (W.L. Gore & Associates) were considered, their increased flexibility made them less suitable for providing the necessary support in this setting. While the endoconduit technique demonstrates high primary patency rates, the potential risk of stent collapse and the need for secondary interventions highlight the necessity of ongoing surveillance to optimize patient selection and procedural strategies.^8^

This case report presents a highly challenging case initially treated with the TA approach TAVI that required subsequent conversion to TF approach TAV-in-TAV. Even in challenging cases such as this, appropriate sheath selection, use of endoconduit, and varied procedural techniques can achieve successful outcomes. Long-term follow-up of the stent graft is planned to assess its durability and identify potential complications, to provide insights into this technique’s long-term feasibility.

## Supplementary Material

ytaf315_Supplementary_Data

## Data Availability

The data underlying this article are available in the article and in its online Supplementary material.

## Lead author biography

Ryo Otake specializes in structural heart intervention and has performed 50 TAVI procedures.
